# Application of Differential Scanning Calorimetry (DSC) and Modulated Differential Scanning Calorimetry (MDSC) in Food and Drug Industries

**DOI:** 10.3390/polym12010005

**Published:** 2019-12-18

**Authors:** César Leyva-Porras, Pedro Cruz-Alcantar, Vicente Espinosa-Solís, Eduardo Martínez-Guerra, Claudia I. Piñón-Balderrama, Isaac Compean Martínez, María Z. Saavedra-Leos

**Affiliations:** 1 Centro de Investigación en Materiales Avanzados S.C. (CIMAV), Miguel de Cervantes # 120, Complejo Industrial Chihuahua, Chihuahua 31136, CHIH, Mexico; cesar.leyva@cimav.edu.mx; 2 Coordinación Académica Región Altiplano, Universidad Autónoma de San Luis Potosí, Carretera Cedral Km, 5+600, Ejido San José de las Trojes Matehuala, San Luis Potosi 78700, SLP, Mexico; pedro.cruz@uaslp.mx (P.C.-A.); isaac.compean@uaslp.mx (I.C.M.); 3 Coordinación Académica Región Huasteca Sur de la UASLP, Universidad Autónoma de San Luís Potosí, km. 5, Carretera Tamazunchale-San Martín, Tamazunchale 79960, SLP, Mexico; vicente.espinosa@uaslp.mx; 4 Centro de Investigación en Materiales Avanzados S.C. (CIMAV), Alianza Norte No. 202, Autopista Monterrey-Aeropuerto Km 10, Parque de Investigación e Innovación Tecnológica (PIIT), Apodaca 66600, NL, Mexico; eduardo.martinez@cimav.edu.mx

**Keywords:** thermal analysis, modulated differential scanning calorimetry (MDSC), phase transitions

## Abstract

Phase transition issues in the field of foods and drugs have significantly influenced these industries and consequently attracted the attention of scientists and engineers. The study of thermodynamic parameters such as the glass transition temperature (Tg), melting temperature (Tm), crystallization temperature (Tc), enthalpy (H), and heat capacity (Cp) may provide important information that can be used in the development of new products and improvement of those already in the market. The techniques most commonly employed for characterizing phase transitions are thermogravimetric analysis (TGA), dynamic mechanical analysis (DMA), thermomechanical analysis (TMA), and differential scanning calorimetry (DSC). Among these techniques, DSC is preferred because it allows the detection of transitions in a wide range of temperatures (−90 to 550 °C) and ease in the quantitative and qualitative analysis of the transitions. However, the standard DSC still presents some limitations that may reduce the accuracy and precision of measurements. The modulated differential scanning calorimetry (MDSC) has overcome some of these issues by employing sinusoidally modulated heating rates, which are used to determine the heat capacity. Another variant of the MDSC is the supercooling MDSC (SMDSC). SMDSC allows the detection of more complex thermal events such as solid–solid (Ts-s) transitions, liquid–liquid (Tl-l) transitions, and vitrification and devitrification temperatures (Tv and Tdv, respectively), which are typically found at the supercooling temperatures (Tco). The main advantage of MDSC relies on the accurate detection of complex transitions and the possibility of distinguishing reversible events (dependent on the heat capacity) from non-reversible events (dependent on kinetics).

## 1. Introduction

This text attempts to be a guide for the reader who wishes to delve into the topic of thermal analysis. Specifically, we want to show the benefits of the so-called modulated differential scanning calorimetry (MDSC). For this reason, we do not go too far into other characterization techniques; nevertheless, we present them so that the reader may have a wider context on the subject. The aim of this work is to show the importance of the modulated DSC in the characterization and identification of the different phase transitions commonly observed in the thermal analysis of food products and pharmaceutics. The text is divided into four sections and the conclusions. These sections are as follows: (1) introduction, (2) phase transitions, (3) comparison of the conventional technique against the modulated technique, and (4) examples of the application of the DSC techniques in different industrial fields.

### 1.1. Phase Transitions

**Figure 1:**
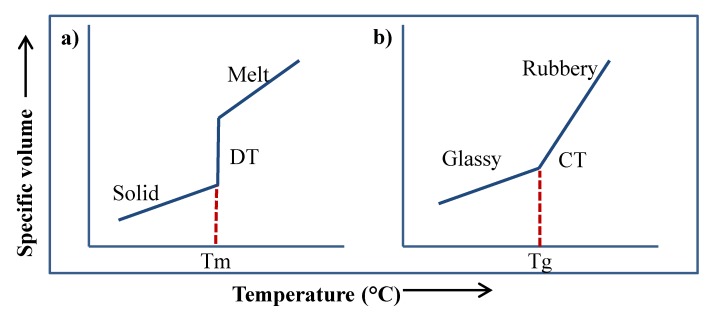
Schematic representation of first and second-order transitions. (**a**) Melting transition of a crystalline solid. (**b**) Glass transition of an amorphous solid.

The transformation of matter from one state to another is called phase transformation. This kind of transition may occur in any of the states of matter such as solid, liquid, or gas. Thermodynamically, the driving force responsible for conducting a phase transition is the chemical potential (µ), which is affected by temperature and pressure [1,2,3]. When there is a gradient in the chemical potential between two phases, a spontaneous phase transition is produced from the higher chemical potential phase to the lower chemical potential phase [4,5]. This transition continuous until the equilibrium is achieved. Thus, in the equilibrium, the chemical potential between the two phases is zero [6]. Overall, phase transitions may be grouped in two types: first-order and second-order transitions [7]. The former comprises the transitions that include a change in some of the thermodynamic properties of the system such as entropy (S), enthalpy (H), or volume (V). These transitions are characterized by the absorption or release of latent heat carried out during the isothermal changes of matter such as liquid–solid, solid–liquid, and gas–liquid [8]. Commonly, first-order transitions include crystallization, melting, condensation, and evaporation. Unlike first-order transitions, in second-order transitions, there is not a change in the latent heat or volume of the system. The thermodynamic properties that are modified during this type of transition include heat capacity (Cp), isothermal compressibility (β), and the coefficient of thermal expansion (α). A second-order transition commonly observed in polymeric systems is the glass transition temperature (Tg), which is defined as the change associated with variations in the molecular mobility and relaxation time in amorphous solids [9]. Tg is a time–temperature-dependent transition carried out by a variation in the temperature, pressure, or humidity of the system, and it is considered as the change in either direction between the rubbery and glassy states [10,11]. However, because of the unbalanced nature of the glassy state and since the vitrification process may take place in a wide range of temperatures, Tg is better identified as a state transition rather than as phase transition [8,12]. Figure 1 shows a schematic representation of these transitions. For example, a first-order transition such as the melting of a crystalline solid is observed as an abrupt change in the specific volume (discontinuous transition, DT), which is isothermally produced by the heat absorption between the system and its surroundings (Figure 1a). Conversely, second order transitions do not involve the abrupt change in the specific volume but rather a continuous change in the slope as the temperature increases (Figure 1b). The temperature at this inflection point is known as the glass transition temperature (Tg). At this temperature, the molecules increase their free volume, having the possibility of creeping over each other. An example of this is the transition of an amorphous material passing from the glassy to the rubbery state.

The study of phase transitions has a large impact on different fields such as the food industry, pharmaceutical industry, and polymers. In food products, Saavedra-Leos et al. [13] suggested that Tg is a useful indicator for understanding the mechanism of food processing and for predicting the life of food products during storage. Ronkart [14] observed the plasticizer effect of water in food products, which is the major component in the product and is responsible for the depression in Tg. In this sense, there are several works reported in literature where the Tg has been determined as a function of water activity (a_w_) or amount of water adsorbed. With the data collected, state diagrams have been constructed for different food-based systems. In pharmaceutics, the understanding of the phase transitions is necessary for characterizing the amorphous and crystalline solid dispersions [15]. A solid dispersion is defined as the active compound dispersed in a solid excipient that may be amorphous or crystalline; e.g., in a crystalline solid dispersion, the crystalline drug is dissolved in a crystalline carrier or excipient. One of the main problems during the development of new drug products is related to establishing the solubility and stability conditions of the active ingredient. In the polymers field, the study of phase transitions has been crucial for understanding the relation between properties and microstructure. Despite the application field, differential scanning calorimetry (DSC) has been the most used technique for characterizing the different phase transitions. However, because different transformations may occur simultaneously during heating, several thermal techniques have been developed as an alternative for conventional DSC. These calorimetric techniques are known as modulated DSC (MDSC) and temperature-modulated DSC (TMDSC) [16].

### 1.2. Phase Transition Characterization by Thermal Analysis

Thermogram is the name given to the curve obtained after running a thermal analysis. Usually, either the temperature or time is plotted on the x-axis, while the acquired signal is plotted on the y-axis. Besides conventional and modulated DSC, among the most employed techniques for thermal analysis are thermogravimetric analysis (TGA), dynamic mechanical analysis (DMA), and thermomechanical analysis (TMA). These thermal analyses and their specific characteristics may be consulted in the literature [17,18,19].

**Figure 2:**
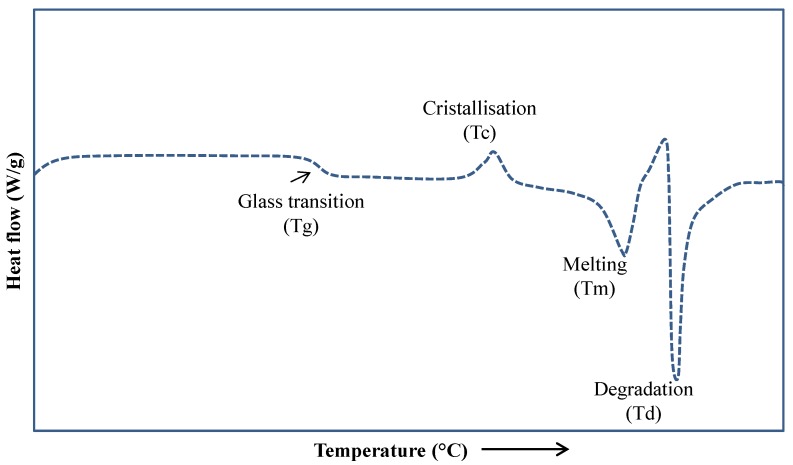
Schematic representation of thermal transitions in semicrystalline material obtained from differential scanning calorimetry (DSC) thermogram [22].

As mentioned before, DSC provides qualitative and quantitative information to the thermal properties of solid materials such as the melting and degradation temperatures, glass transition temperature, melt and crystallization enthalpy, specific and latent heats, polymorphism, and purity of the materials [20]. Briefly, the method comprises supplying heat at a constant rate to the sample and a reference material. The difference in the heat flow necessary to supply into the sample and the reference material in order to keep both at the same temperature. This heat flow difference is plotted against the temperature or time for obtaining a typical thermogram, as shown in Figure 2. The thermogram shows four transitions identified from low to high temperature as: glass transition temperature (Tg), crystallization temperature (Tc), melting temperature (Tm), and degradation temperature (Td). More complex thermal transitions such as solid–solid (Ts-s), liquid–liquid (Tl-l), vitrification, and devitrification temperatures (Tv and Tdv, respectively) could be obtained by widening the range of temperatures of the thermal analysis and reaching the supercooling temperatures (Tco) [21].

In general, first-order transitions are observed as well-defined peaks, while second-order transitions are variations in the heat flow curve. Tg is observed as a slight change in the slope of the curve. As the temperature increases, the material gains enough energy and the microstructure is reorganized, observing an exothermic peak at Tc. At a higher temperature, the system has gained so much energy that the separation between the molecules is large enough to break the intermolecular interactions required for keeping the molecules together. The system lowers its viscosity and melts at Tm. The addition of further energy at higher temperatures produces the oxidative decomposition and degradation processes observed at Td. Additionally, modulated DSC is employed in the thermal characterization of complex thermal events, which are not clearly observed by conventional DSC [11,23,24]. The correct interpretation of the heat flow curve is not trivial when several thermal events are presented at the same temperature interval. Different transitions may overlap for a single component material, e.g., the recrystallization and melting of a semicrystalline material. Thus, for a multicomponent material, the correct identification of the transitions is more complicated [24].

**Figure 3:**
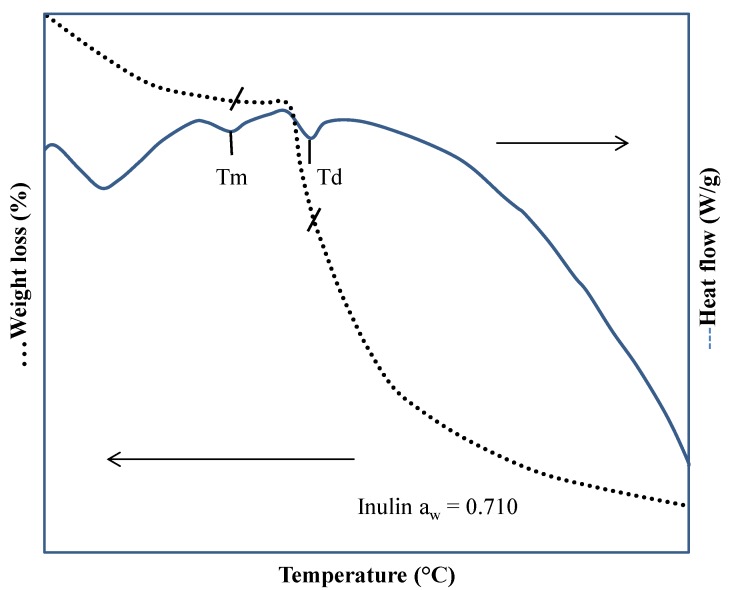
Thermogravimetric analysis (TGA)-DSC thermogram of inulin obtained from Dalia at water activity aw = 0.071 and a heating ramp of 5 °C/min [22].

TGA is mainly employed for determining the mass loss when a sample is heated, cooled, or keep at a constant temperature in a controlled atmosphere. Its application is dedicated to the analysis of products in the quantification of volatiles, degradation of matter, combustion reactions, and residual matter. With TGA, it is possible to study the decomposition of organic materials and observe the progress of the individual components. The simultaneous TGA-DSC allows acquiring two signals (mass loss and heat flow) in a single experiment. With the simultaneous TGA-DSC, it is possible to differentiate the melting from the degradation when these events occur in a narrow range of temperatures (e.g., in sugar-rich systems); the melting and degradation temperatures are largely affected by the amount of water adsorbed. Thus, the determination of the actual value of these properties is difficult, because the events may overlap or the signal may be very low to be properly noticed. Figure 3 shows a typical simultaneous TGA-DSC curve obtained from inulin from Dahlia tubers at a water activity of 0.71. As observed in the heat flow curve, the melting event identified as the Tm peak is unclear and difficult to appreciate. The degradation temperature identified as Td is observed at a higher temperature and may be confused with the melting. The TGA curve shows that at the temperature of Td, the event is accompanied by a stepped mass loss, while at Tm, the mass loss curve is flat. With this type of thermal analysis, Saavedra-Leos et al. [22] determined the initial, maximum, and final melting and degradation temperature ranges for inulin at different water activities.

**Figure 4:**
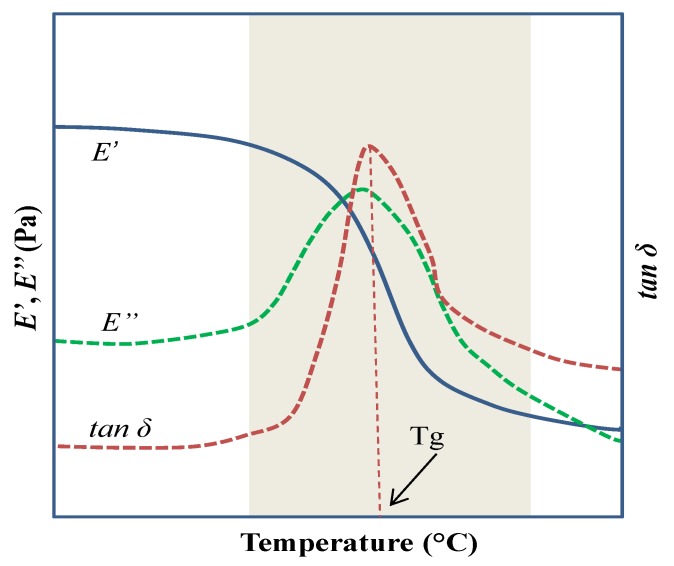
Typical DMA thermogram showing the evolution of E’, E”, and tan δ as a function of the temperature [11].

Unlike DSC and TGA, where powders may be analyzed, the conventional DMA measures the mechanical properties of viscoelastic solids and films subjected to a constant oscillatory force or deformation. Commonly, the results obtained from DMA include three signals plotted against temperature, time, or frequency. The signals correspond to the curves of the storage modulus (E’), loss modulus (E’’), and the damping factor (tan δ). This technique is preferred over DSC to determine the Tg of solid materials. With this technique, Tg is reported as a temperature range rather than a single value. However, the main disadvantage when analyzing food products is that usually these products are powder-based, which requires further processing in order to form a continuous solid film [25]. Additionally, the determination of Tg by DMA requires the correct selection of the geometry used for setting the sample and the availability of a temperature chamber that is able to withstand the extreme cooling and heating conditions. Figure 4 shows a typical DMA curve employed in determining the Tg of a polymer. Tg is determined from the temperature range comprising a sudden decrease in E’ and the maximum values in both E’’ and tan δ.

**Figure 5:**
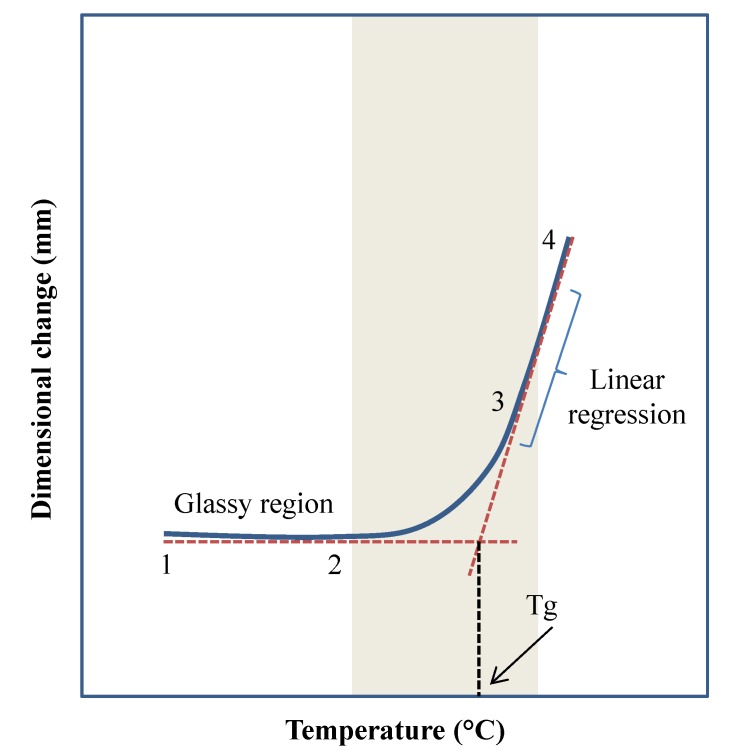
Thermal mechanical compression test (TMA) to determine transition temperature. The glass transition temperature (Tg) is considered as the temperature where a sudden dimensional change occurs [11].

TMA is utilized for determining the dimensional changes of solid samples under controlled conditions of temperature, time, load, and atmosphere. These changes include expansion, penetration, tension, compression, and deformation. The type of the sample may include powders, fibers, coatings, and thin films. Some of the properties determined by this technique are expansion, penetration, tension, compression, and strain. It is also useful to determine transitions such as Tm and Tg, the thermal expansion coefficient and the softening point, as shown in Figure 5.

## 2. Modulated Differential Scanning Calorimetry (MDSC)

**Figure 6:**
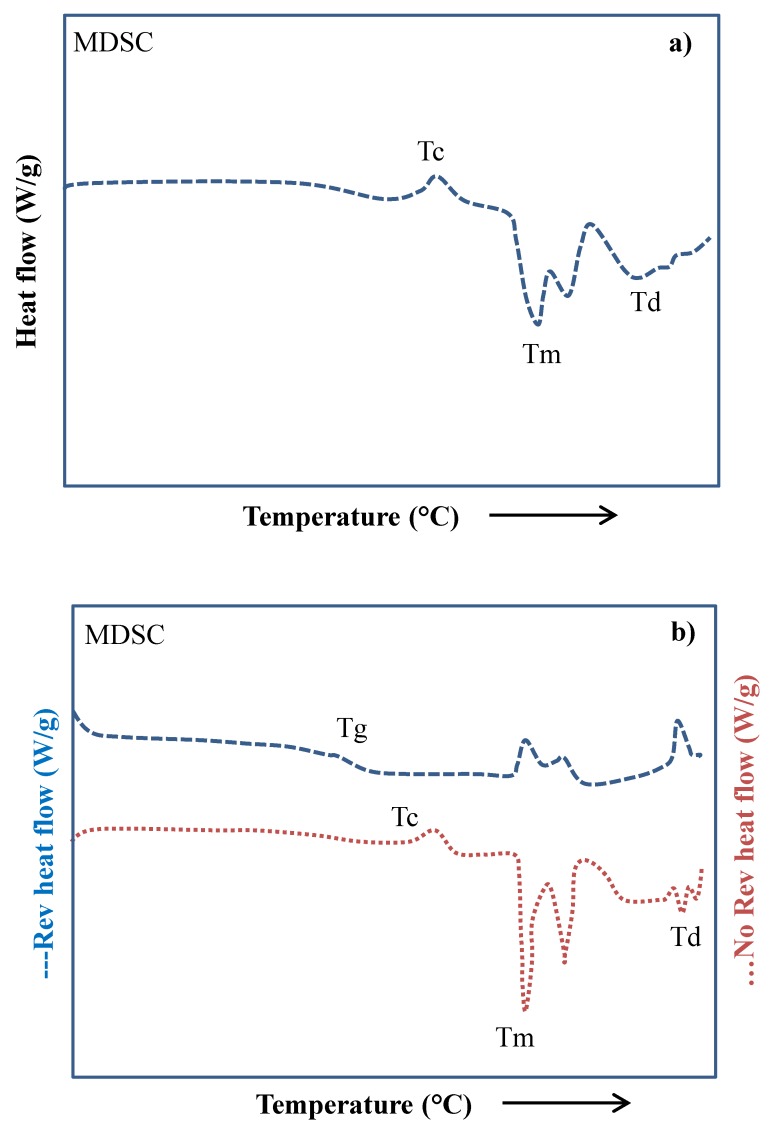
(**a**) Modulated DSC (MDSC) thermogram of the total heat flow of Inulin. (**b**) MDSC heat flow thermogram separated in two components: reversible and non-reversible events used for the identification of different transition temperatures of Inulin such as Tg, Tc, Tm, and Td [22].

Some of the main advantages offered by conventional DSC over other thermal characterization techniques include the wide range of temperatures (−90 to 550 °C) where transitions may be observed, and the ease for analyzing the data with the adequate software. The correct interpretation of results is difficult to carry out sometimes, and even more in systems with mixtures of similar components [24]. The main problem observed in this type of systems is the overlapping of some phase transitions. This problem is caused by the instrumentation of the technique, because heat flow is acquired as the average of several heat flow signals. The DSC measures the temperature differences in the heat flow of the sample and a reference material at a constant heating rate, and detects differences in the heat flow (ΔQ) between both materials. One solution to this problem is the use of larger amounts of sample and the decrease in the heating rate [11]. Ozmen and Langrish [26] studied the effect of heating rate on the Tg of skim milk powder with a conventional DSC. They noted a better resolution in the thermogram at a higher heating rate and a decrease in glass transition temperature from 50.3 to 46.2 °C when the heating rate decreased from 10 to 2 °C/min. Other difference between these modes of operation is the determination of heat capacity (Cp). With MDSC, Cp is directly determined even at very low heating rates. While in conventional DSC, the determination of Cp involves running three experiments: first with the empty cell, the second with a reference material, and finally with the sample. MDSC technology allows the direct measurement of heat capacity, even at very low heating rates (quasi-isothermal) conditions, thus simplifying the experimental work and making the measurement more accurate. In this sense, MDSC was developed to overcome the disadvantages of conventional DSC. MDSC employs simultaneously two heating rates: a linear ramp that provides the same information as the conventional DSC, plus a sinusoidal heating rate that allows differentiating between the reversible thermal events dependent on heat capacity from the non-reversible events related to kinetics changes such as melting, crystallization, and curing reactions [27]. MDSC takes advantage of the modulation of temperature for calculating the heat capacity of the sample during the same experiment, while it does not require cooling during modulation. The modulated heat flux signal is measured during the experiment and is used to calculate the signals. Three signals are acquired during the MDSC: total, reversible, and non-reversible heat flow. Since the glass transition phenomenon involves a change in the specific heat capacity of the material, the signal corresponding to this event is the reversible heat flow. In the non-reversible heat flow curve, the thermal events comprise a kinetic component and are observed as peaks that involve an enthalpy change such as thermal curing reaction, cold crystallization, or vaporization. The MDSC is capable of recovering data in a single measurement that can be separated into two signals, heat capacity and kinetic components. An example of the use of MDSC in the field of food products was reported by Saavedra-Leos et al. [22]. They studied the thermal properties of inulin, which is considered to be a biopolymer. With the MDSC, the total heat flow curve was separated into two contributions: reversible and non-reversible. Figure 6a shows the total heat flow curve for inulin, while Figure 6b shows two contributions of the total heat flow curve (reversible and non-reversible). In the total heat flow curve, different transitions are observed that are identified as Tc, Tm, and Td, but Tg is not observed. In the reversible heat flow curve, Tg is clearly observed together with some other molecular rearrangement events. In the non-reversible flow, the heat flow curve is better defined through the kinetics events Tc, Tm, and Td. The sum of the reversible plus the non-reversible flow is the total heat flow. Saavedra-Leos et al. [28] reported the study of commercial maltodextrins with different dextrose equivalents. The Tg of each maltodextrin was identified by MDSC measurements, and the results showed a decrement of 70–80 °C in all of the cases. The abrupt change in Tg was attributed to the plasticizing effect caused by the formation of hydrogen bonds between water molecules and the hydrophilic glucose segments in maltodextrins. Later, Araujo-Diaz et al. [29] evaluated the physical properties and conservation of antioxidants in blueberry juice employing carrying agents as aids in the spray drying of the juice. The powders obtained were subjected to different levels of water adsorption, and Tg was identified from MDSC measurements. The results evidenced a decrease in Tg as water activity increased. They also conclude that both carbohydrates polymers were effective as carrying agents during the spray-drying process of blueberry juice; however, maltodextrin showed better performance in terms of the antioxidants conservation. Leyva-Porras et al. [30] studied the physicochemical and thermal properties and the effect of degree of polymerization (DP) of inulin. MDSC was employed in order to determine thermal events such as Tm, Tg, and Td. Inulin with higher DP showed higher water content, since a large number of hydrogen bonds were formed. With the adsorption of water, chain mobility was promoted, directly affecting the melting, degradation, and glass transition temperatures. Tg showed a decrease and wider temperature range as the water content increased. Additionally, high DP inulin showed a higher melting range and degradation temperature than low DP inulin. Other applications fields of MDSC have been the evaluation of enthalpy of relaxation at Tg (ΔHnr) and the melt fragility of some inorganic systems.

## 3. Supercooling Modulated Differential Scanning Calorimetry (SMDSC)

Freezing is widely used and an efficient mode of preserving food quality and prolonging the shelf life of food products. By reaching supercooling temperatures, it is possible to greatly reduce a large number of chemical reactions and microbial growth responsible for the deterioration of foods properties such as loss of texture, color, and taste. By definition, supercooling or undercooling is the process of lowering the temperature of a liquid or gas below its freezing point, without changing its initial phase i.e., the formation of ice crystals. The temperature at which supercooling is reached is termed as Tc. The identification of this thermal event is usually carried out in a modulated DSC equipped with a liquid nitrogen cooling system (LNCS) or other inert gases that are capable of lowering the temperature below −160 °C. During the obtaining of this thermal event, other commonly observed events include Tl-l, Ts-s, Tv, and Tdv. A Ts-s is a first-order structural transition carried out in the solid state, which is commonly observed in crystalline solids and can be understood as the transition of a crystalline structure into a different crystalline structure, an amorphous solid, or vice versa. The Tl-l phase transition is a transformation of a liquid into another one with different density and entropy values. This transition is regularly observed in mixtures; however, it has also been observed in pure substances [31].

If the temperature decrease is sufficient to exceed the freezing point, a series of microstructural changes related to the formation of ice crystals may take place, and consequently, the physical and chemical properties may be affected. The freezing rate is the most important parameter controlling the crystallization and the resulting microstructure, since crystal size and morphology are highly modified [32]. At high freezing rates, the agglomeration rate of water molecules is higher than the dissociation rate; therefore, the nucleation process is greatly favored, promoting the formation of a large number of small crystals. Slow freezing rates promote the slow removal of heat from the system, decreasing the nucleation rate, which is observed as the formation of some large ice crystals. According to Fukuma et al. [33], although the formation of ice crystals is undesirable, and since a supercooled product is unstable, the nucleation and crystallization may occur at any time. In this sense, the study of thermal properties at supercooled temperatures may provide helpful information about the storage conditions that may minimize the crystallization process or promote the formation of a large amount of small and well-distributed ice crystals. MDSC at supercooling conditions (SCMDSC) is of great help for this task, since it allows operating in a wide interval of temperatures i.e., −160 to 50 °C where complex thermal events such as Tl-l and Ts-s are usually observed. The vitrification temperature (Tv) is defined as the temperature at which the transformation of a substance into a glassy state occurs in the form of a non-crystalline amorphous solid. In contrast, the devitrification temperature (Tdv) corresponds to the temperature at which crystallization in a formerly crystal-free glass solid occurs.

**Figure 7:**
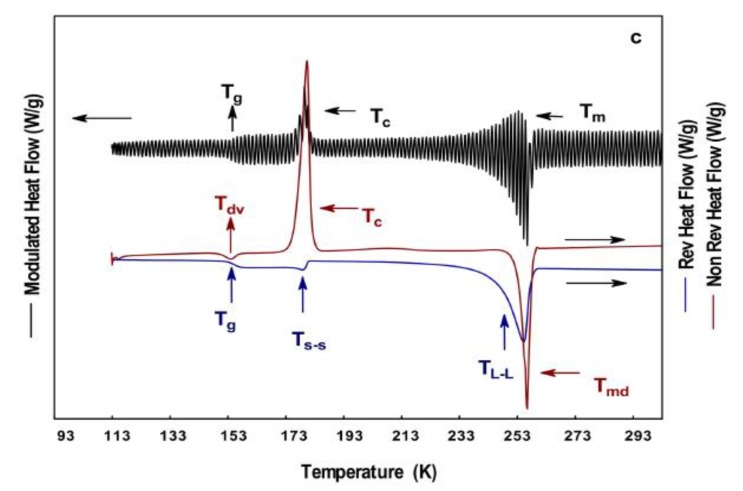
Supercooling MDSC (SMDSC) thermograms of ethylene glycol at 278 K/min: (**a**) DSC, (**b**) MDSC, and (**c**) SMDSC [21].

The supercooling study is relevant for materials presenting phase transitions at relatively low temperatures, such as the polyols (glycerol, sorbitol, ethylene glycol, propylene glycol, poly (ethylene) glycol, and poly (propylene) glycol). Although it is possible to identify the mentioned complex thermal events with a regular DSC coupled to a supercooling system, it is not possible to differentiate the reversible from the non-reversible, since only one signal is acquired. In this sense, supercooling modulated DSC overcomes this issue. Recently, Toxqui-Terán et al. [21] employed calorimetric techniques (DCS, MDSC, and SCMDSC) for the complete thermal characterization of glycerol, ethylene glycol, and propylene glycol. With the aid of these techniques, they differentiated regular from complex thermal events. From the three polyols, ethylene glycol showed six reversible thermal events identified as Tg, Ts-s, and Tl-l, and three non-reversible events identified as Tdv, Tc, and Tm (Figure 7).

## 4. Applications of DSC and MDSC in the Industrial Processing and Conservation of Matter

### 4.1. Food Products

Food products are defined as multicomponent systems where the individual constituents may experience phase transitions in the range of temperatures and pressures at which the products are processed, stored, and consumed [2,34]. The molecular changes exerted during the phase transitions promote variations in the thermal, mechanical, and transport properties of the products. Thus, the study of phase transitions is of major importance for the adequate control, distribution, and storage of food products [2]. Water is one of the most important components in fresh and liquid foods. The state of aggregation of water defines the physical state of the food product, where water may be found as solid, liquid, or vapor at the typical temperatures of processing, and storage [2,34]. For food products with low water content or a high content of solids, phase transitions in proteins, carbohydrates, and lipids also play a major role in the final properties of these products [12,35]. In this regard, DSC has been extensively used for analyzing the phase transitions of food products. When analyzing the proteins contained in food products, DSC is mainly used to determine first-order transitions such as the denaturation of proteins. This process comprises the reversible or irreversible loss of the original ordered structure into a disordered arrangement of the polypeptide chains. The denaturation temperature is defined as the temperature where at least 50% of the structure of proteins has been lost [36,37,38]. Once proteins have been denatured, the biological and physical properties are lost, presenting changes in the solubility, viscosity, and diffusion coefficient. Sanchez et al. [39] studied the effect of storage conditions on the denaturing of corn proteins. They found that after 30 days of storage, polar proteins such as albumin and globulin were affected in 80% of the products, while after 50 days, hygroscopic proteins such as prolamin and glutelin were affected in the same proportion. Zhang et al. [40] reported endotherms of soluble proteins at 70 and 90 °C for the denaturation β conglycinin and glycinin proteins, respectively. Rastegari et al. [41] studied the thermal stability of pepsin under four different acidic conditions (pH 1, 2, 3, and 4). They analyzed the DSC profiles at which they observed the stability of the protein in the different conditions through the relationship between the calorimetric enthalpy (ΔHcal) and the van’t Hoff enthalpy (ΔHVH), which is considered as a measure of the denaturation ability of the protein. Finally, they concluded that proteins at a pH of 4 showed greater stability, presenting the lowest ΔHcal/ΔHVH ratio, which indicated a reduced ability to denaturation. Furthermore, Frydenberg et al. [42] used the DSC to assess the impact of high-density ultrasound (HDU) on the thermal properties of whey proteins, which are widely used in food materials due to the ability of forming thermo-induced gels to enhance texture and water retention in the food product. HDU-treated proteins were analyzed immediately by DSC, finding that denaturation was associated with the disruption of the intramolecular bonds identified as endothermic processes in the thermograms. Additionally, it was possible to relate the changes in the denaturation enthalpies with changes in bond patterns, where the conformational state of the protein with weak bonds may require less energy to arrange and therefore cause a reduction in enthalpy. Górska et al. [43] obtained b-lactoglobulin and vitamin D3 complexes by using spray drying at relatively high temperatures (120–150 °C). Subsequently, they studied the thermal properties of the complexes prepared based on b-lactoglobulin–vitamin D3 and b-lactoglobulin–vitamin D3–lactose. MDSC was employed for evaluating the effect of the spray-drying methodology on the calorimetric parameters. The thermograms showed an increase of 9.25 °C in the temperature of denaturation of b-lactoglobulin attributed to the binding of vitamin D3, which increased the resistance and stability of the protein. Escobedo et al. [44] presented a novel methodology employing MDSC for the detection and quantification of liver fibrosis. Liver fibrosis was induced on male Wistar rats by the use of two experimental procedures including common bile duct ligation (BDL) and carbon tetrachloride administration (CCl4). The level of fibrosis was categorized in three forms: mild grade of hepatic fibrosis (F1), intermediate degree of disease (F2), and advanced stage of fibrosis (F3–F4). Depending on the stage of disease, the hepatic tissue exerted a typical denaturation. Thermograms of the total heat flow, non-reversible heat flow, and heat capacity were analyzed in detail in the range of 70 to 90 °C, and significant differences in heat capacity were observed. Control samples (exerted liver without any level of fibrosis) exhibited a transition temperature onset of 95 °C. In contrast, at the F1, F2, and F3–F4 fibrosis stages, the transition temperature significantly decreased to 93 °C, 84 °C, and 75 °C, respectively. Likewise, the number of transitions concerning the heat capacity simultaneously increased with the grade of fibrotic damage. Morel et al. [45] evaluated the thermodynamic stability of proteins by DSC. They studied the melting of amyloid fibrils of the N47A mutant of the α-spectrin SH3 domain. Fibrils were produced by incubation of the fresh native protein, and according to transmission electron microscopy (TEM) results, curly filaments and thicker fibrils were obtained. Through the DSC analysis, a single endothermic peak was observed, which was attributed to the homogeneity of the thermal behavior despite the heterogeneity in the fibrils’ length. The authors compared the specific melting enthalpy and the changes in the apparent heat capacity of the fibrils against those of the compact globular forms. They concluded that these parameters were significantly lower in the protein fibrils because of the lower density of interactions and the higher area of the hydrated surface. Savadkoohi and Farahnaky [46] made use of MDSC for the thermal study of the heat-induced gelation process of tomato-seed proteins (TSP) and their structural aspects. MDSC was employed for examining the effect of heat treatments on the TSP. The results showed that structural and conformational changes occurred in the material and were associated with the weight percent of TSP in the evaluated solutions. An endothermic peak corresponding to the denaturation temperature was observed and related to the solids content. Additionally, the changes in enthalpies at different solid concentrations (1–10%) were significantly different, suggesting that proteins required more energy for unfolding at high concentrations of TSP.

**Figure 8:**
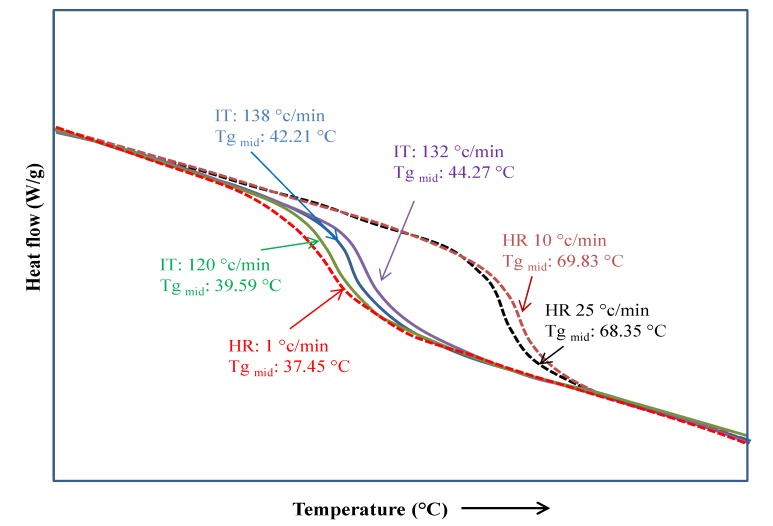
Tg of sucrose obtained from conventional DSC at heating ramps of 1, 10, and 25 °C/min and obtained from MDSC at heating ramps of 120, 132 and 138 °C/min [49].

In the carbohydrates field, Lee et al. [47] studied the effect of heating conditions in the Tg of amorphous sucrose. As observed in Figure 8, they employed three heating rates in conventional DSC and three temperatures in MDSC. From the conventional DSC, the determined values of Tg and Tm increased with the heating rate, while from MDSC, the Tg values were very similar for the three tested temperatures. Recently, Knopp et al. [48] published a review of the recent advances and the potential applications of MDSC in the development of drugs. Additionally to the study of different physicochemical properties or solid–solid and solid–liquid transitions, they included modern applications related to the field of biophysics. For example, Tg was determined to be a key parameter in the embryogenesis and silk proteins, where MDSC was employed to differentiate the structural changes adopted by proteins occurring at the glassy state from those induced by the presence of water molecules.

In complex carbohydrates, polysaccharides have been widely used as carrying agents because of their relative high Tg. These compounds are mixed in proportions of 10–30% and dried to obtain functionalized powdered foods. The carrying agent protects the active ingredient from thermal decomposition and the environment, extending the shelf life of the product. Hinrichs, Prinsen, and Frijlink [50] employed the MDSC to determine the Tg of inulin when used as a carrier agent in the stabilization of proteins during the spray-drying process. They reported that the Tg of inulin is beneficial for the drying process and the storage conditions of the protein product. Ronkart et al. [14] determined the Tg and the formation of crystals of inulin subjected to different conditions of humidity. At a water activity of 0.56, the Tg of inulin was lower than the average storage temperature, which produced the collapse of the amorphous structure of inulin particles during storage. Additionally, Saavedra-Leos et al. [22] proposed the use of inulin as a carrier agent and nutrient in a functional food product. Based on the concepts of Tg and water adsorption, they set the optimal storage conditions of the product below a water activity of 0.21 and temperature of 47 °C. At these conditions, the crystalline structure of inulin and morphology of the particles may remain intact i.e., amorphous and with a spherical shape. Vuddanda et al. [51] studied the effect of different plasticizers (glycerol, vitamin E, and triacetin) at different concentrations (10%, 20%, and 30% *w*/*w*) on the physicomechanical properties of pullulan-based oral films. MDSC was employed in the determination of Tg of the pullulan films produced by a casting solvent process. They found that glycerol reduced the glass transition temperature of the pullulan biopolymer from 222 to 116 and 74 °C in with 20% and 30% of the plasticizer, respectively. The authors concluded that the presence of the plasticizer not only favors the manufacturing process of the film by lowering the minimum film forming temperature, but it also facilitated the formation of soft and flexible films.

In lipids, which are heterogeneous and complex compounds, thermal analysis is mainly focused on determining the first-order transitions such as the transformation of the crystalline structures (sub α, α, β and β’), the melting, and the second-order transitions to set the crystalline structures (sub α and α). The importance of understanding these transformations is that in some food products, the crystalline α structure is unstable, while the β form is reached at very high temperatures. Additionally, the melting temperature in lipids is related to the type of lipid and processing. Therefore, several authors have reported different crystallization profiles, ranges of melting temperatures, and polymorphic forms [52,53,54,55,56]. Tolstorebrov et al. [57] studied the thermal transitions by DSC and MDSC of 18 triglycerides and 4 fish oils in a temperature range of −150 to 80 °C. From the MDSC, they distinguished the Tg and the melting of α, and β crystals in the reversible heat flow curve, while in the non-reversible curve, they observed a kinetic event called cold crystallization. Afoakwa et al. [52] characterized the melting temperature by DSC of chocolate, and related its particle size and composition (based on the percentage of lecithin and fat added) with the crystallization of the product. In chocolate products, crystallization is undesirable and is considered as a storage fault, since the crystals are perceptible to taste and harden the product. From the results, it was established that the chocolate particle size does not affect the crystallization, but it does affect the amount of fat added. Conversely, the addition of lecithin reduces the formation of crystals. In novel applications such as 3D food printing, the study of the thermal properties and phase transitions is of great interest in the determination of optimal printing conditions. The DSC analysis is crucial for the understanding of the processing conditions and optimization of shape, color, taste, texture, and nutritional composition [58,59,60]. For example, Mantihal et al. [61] studied the 3D printable conditions of chocolate adding 5% of magnesium stearate (Mg-St) as a lubricant enhancer. They found that Mg-St modified the flow (viscosity) and thermal properties (Tm and Tg). While the extrusion characteristics of the final 3D-printed chocolate remained, the addition of Mg-St did not alter the thermal properties of the chocolate but delayed the crystallization process. Cañadas and Casals [62] published a chapter providing a full explanation of the thermotropic properties of protein–lipid interactions, discussing in detail the necessary requirements in order to optimize the protocols for sample preparations, analysis, and interpretation of the data of lipid-phase transitions.

Supercooling is a food processing technique that has been extensively studied since it has the potential of increasing the shelf life of food products [63]. The process uses storage temperatures below the initial freezing point of foods while maintaining the quality characteristics associated with fresh foods such as color, texture, and taste. Jeremiah and Gibson [64] investigated the effects of different atmospheres, temperatures, storage times, and packing methods of 216 pork loin sections. They found that lower storage temperatures (−1.5 °C) increased the length of the acceptable appearance and reduced the number of unacceptable sample scores. The pork meat stored at −1.5 °C also showed significantly fewer smells in comparison with those kept at higher storage temperatures (2 and 5 °C).

### 4.2. Pharmaceutical Products

The pharmaceutical industry spends considerable time and money on developing and testing new methods to ensure the final quality of products [65], determining the concentration and distribution of active pharmaceutical ingredient (API) or drug components, and determining the matrix of the pharmaceutical product or excipients [66]. According to Patel [67], approximately 90% of the pharmaceutical products available in the market are in the solid form (e.g., tablets), so its components (API and excipient) may be present in a crystalline or amorphous solid state. The crystalline solid is characterized by its rigidity and long-range order, where the atoms occupy specific positions in the network. The amorphous solid is referred as a meta-stable state whose molecules have a disordered structure and whose volume is larger than the crystalline system, even with the same chemical composition [2,68]. Although the structural arrangement in the amorphous solid is disordered in some areas, it may have a short-range molecular order similar to a crystalline structure. However, a similar order only occurs in molecular dimensions and in the range of a few angstroms [8]. The API is the substance or drug that is chemically or biologically active. The API is a solid dispersed in an excipient; the API may be crystalline or amorphous, while the excipient may be solid, liquid, or gas. In the pharmaceutical products, it is important to consider the initial structure of the API, since an amorphous solid presents major physical stability that is observed as the changes in the solubility and dissolution rates [69]. A crystalline solid dispersion is the name given to the products containing a crystalline API dissolved in a crystalline excipient. The different ways for combining the dispersion are employed to set the conditions of the solubility and stability of the API. In this sense, DSC is an excellent tool to characterize the solid dispersions, where Tg has been widely employed in the prediction of the stability of these products [15]. Pharmaceutical solid dispersions comprises a mixture of two amorphous solids differing in chemical composition or a crystalline drug dispersed in an amorphous matrix. In this type of product, it is desirable that the initial state of the drug remains unchanged during the lifetime of the product. Then, the correct determination of the Tg may be helpful for setting the storing conditions for avoiding phase transitions. Thus, in order to avoid undesired physical changes such as crystallization and chemical reactivity, it is very important to identify the conditions at which the molecular motion processes begin. Shamblin et al. [70] characterized the Tg of amorphous systems (indomethacin, sorbitol, sucrose, and trehalose) for setting the molecular relaxation without exceeding the storage time. They found that some molecular processes such as crystallization and chemical reactivity are related to the molecular rearrangement of the amorphous structure with time, and that the molecular structure was better preserved when the product was stored at temperatures below the Tg.

The correlation between the stability of dispersions and Tg is often used to make judgments about the probable physical stability of amorphous systems. However, it is still difficult to predict a relationship between Tg and the stability of dispersions, since for pure amorphous solids, the Tg is unreliable in relation to the physical stability of the system. Baird and Taylor reviewed the thermal analysis to explain the properties of solid pharmaceutical dispersions, which may comprise two phases: two amorphous phases of different composition or crystalline drug particles in an amorphous matrix. The overall state of the drug is an amorphous solid with improved dissolution characteristics and good physical stability. In order to achieve a greater physical stability, the thermodynamic parameter Tg may be useful in establishing the physical behavior of the pharmaceutical systems, since in the formulation of dispersions, there is regularly more than one component, which increases the complexity of the systems. Therefore, the accurate determination of Tg of the solid dispersions may provide relevant information related to the physical properties of the system, the physical state (amorphous, semi-crystalline, or crystalline) of the drug/carrier, the miscibility of the drug into the carrier, and the storage conditions.

The determination of the crystallinity in the pharmaceutical industry is very important in the quality control of raw materials, intermediate products, and final products, and to test the effectiveness of technological procedures for processing control. Several characterization methods are commonly employed to study the crystallinity of pharmaceutical solids. These methods include X-ray diffraction (XRD), the determination of the solid-state density by nuclear magnetic resonance (NMR), and the adsorption of water vapor. Additionally, the changes in crystallinity can be detected using DSC and isothermal calorimetry techniques [71]. For example, Smith et al. [72] conducted a DSC thermal study to quantify the residual crystallinity in grinding processes in ball mills of α-lactose monohydrate used as a carrier. Commonly, the quantification of crystallinity in a semicrystalline material involves the comparison of the area under the curve of the melting endotherm against the area under the curve for the melting endotherm of a fully crystallized sample. However, the presence of an amorphous phase in a ground material may affect the final shape of the thermogram by modifying its baseline and the occurrence of other thermal exothermic events of less magnitude such as a devitrification event that contributes to the overall melting enthalpy. Therefore, during the analysis of the DSC curve, it is necessary to subtract this contribution from the crystallization enthalpy in order to have a more accurate estimation of the crystalline content in the ground material [73].

The pharmaceutical industry also requires the development of surgical materials and clinical instruments with improved performance. De Camp [74] presented the regulatory considerations in crystallization processes for bulk pharmaceutical chemicals. In this work, it was stated that at least one validation process is required to verify the polymorphic form of a new drug. Additionally, in the development of a new drug application, it is mandatory to show how the polymorphic form changes the dissolution and bioavailability of the properties. In this sense, the study at a molecular level of the control of the crystallization and phase transitions in the pharmaceutical field has been widely reported [75,76,77,78,79].

The study of pharmaceutical products at supercooling temperatures is relevant for the identification of thermal events presented during the freezing that could affect the performance of certain drugs. For example, lowering the temperature may favor the conditions for the crystal growth of a specific organic compound, which in turn will produce significant problems with parental suspensions. Another example is the phase transition of suppositories made from theobroma oil. When this oil is heated at 60–70 °C and rapidly chilled at supercooling temperatures, it undergoes a polymorphic transition from the α form to β1 and β forms. The latter crystalline structures exhibit higher melting points and can be easily handled [80].

Different interactions at the molecular level result in different phases at the macroscopic level that influence the compound behavior including solubility, bioavailability, and stability [81]. Jadhav et al. [82] described the importance of the glass transition temperature as a tool in the modification of the physical properties of drugs. When a solid is heated and subsequently quenched at supercooling temperatures, molecules are kinetically trapped and instead of crystallizing, the solid is converted in an amorphous material with a glassy appearance. Since the molecule’s movement becomes restrung to only vibrational movements and is not rotational or translational, the Tg is drastically modified, and the physical properties are modified as well. For a material in the solid amorphous state, the behaviors related to the dissolution, bioavailability, processing, and handling are all improved. Yu [83] published an extensive work about the importance of the disordered structures in amorphous pharmaceutical solids, including their preparation, characterization, stabilization, and physicochemical properties regarding the corresponding crystalline forms. Moes et al. [84] prepared ternary solid dispersion with different contents of docetaxel, polyvinylpyrrolidone (PVP)-K30, and sodium lauryl sulfate (SLS) in order to overcome the solubility and dissolution issues of the docetaxel anticancer drugs. MDSC was used in combination with X-ray diffraction (XRD) and Fourier transform infrared spectroscopy (FTIR) to examine the physical properties and find an explanation for the solubility behavior of the samples. The docetaxel/carrier/surfactant weight ratio of 1/9/1 showed the best performance regarding solid dispersion, which means a higher solubility and dissolution rate compared to pure drug and physical mixture formulations.

MDSC has been also employed in the determination of other thermal properties rather than Tg and Tm, such as heat capacity and thermal conductivity. Murdande et al. [85] studied the thermal properties of indomethacin using MDSC. The heat capacity of indomethacin was determined for the crystalline and the amorphous forms of the drug. The heat capacity of the amorphous was higher than its crystalline form in all temperatures, and the heat capacity increased significantly as the amorphous form approached the glass transition. The thermal properties were used in the calculation of the free energy difference between amorphous and crystal forms of indomethacin, which in combination with other measurements provided information for the theoretical prediction of solubility. Baghel et al. [86] studied the tendency and kinetics of crystallization of two amorphous active pharmaceutical systems, dipyridamole (DPM) and cinnarizine (CNZ). MDSC was employed for obtaining the heat capacity changes at the glass transition temperature in order to get the thermodynamic fragility (mT) of both systems. Additionally, other parameters such as the dynamic fragility (mD), heating rate dependence of Tg (mDTg), the mean relaxation time, and the glass-forming ability (GFA) were calculated in order to establish the relevance of these parameters to the crystallization of amorphous drugs. The thermodynamic fragility of DPM and CNZ was calculated as 1.09 and 1.14, respectively, revealing the fragile nature of the active pharmaceutical ingredients (<1.5). They found a significant correlation between mT, mDTg, and GFA, suggesting that fragile glasses have poor GFA and hence a higher crystallization tendency. They concluded that parameters such as fragility, GFA, and crystallization kinetics are important in the prediction of the life expectancy of amorphous drugs. Gunaseelan et al. [87] presented a quantification method using MDSC to determine the amorphous content in crystalline Cefuroxime axetil. The method was based on the linear response of the specific heat determined at the Tg of the amorphous content. The study included the analysis of different amorphous contents (1%, 2%, 5%, 7.5%, 10%, and 100%), and the determination of the corresponding heat capacities (0.005604, 0.01574, 0.02848, 0.04998, 0.06988, and 0.4675 J/g °C), respectively. Additionally, the authors compared the results obtained by MDSC with those from X-ray diffraction and concluded that XRD exhibited poor detection of low amorphous content (<10%), while with MDSC it was identified at a lower percent (>1) due to the sensitivity for Tg determination. Lin et al. [88] reported a method for measuring the thermal conductivity of small-molecule organic solid materials using MDSC. The experimental section involved the compaction of 18 pure small molecule compounds such as acetaminophen, aspirin, carbamazepine III, Υ-indomethacin, and ketoprofen, among others. The powders were consolidated in 6 mm evacuable pellets. The method indirectly obtained the thermal conductivity (k) of pharmaceutical solid materials by the evaluation of the specific heat capacity and the apparent heat capacity, and it was as an alternative of the regular determination of k with MDSC. However, they concluded that sample preparation was imperative i.e., pellets conformation, since the pore volume and powder surface area may affect the accuracy of the results.

Drug–excipient interactions may lead not only to several chemical reactions but also to other physicochemical changes that may reduce the active pharmaceutical ingredient effect [89]. These interactions are termed as incompatibilities. Thus, the study of the compatibility in pharmaceutics is of great importance as well. Yu et al. [90] studied the compatibility of drug–polymer systems (acyclovir–polyacrylonitrile) with different loading ratios. The compatibility was determined by means of conventional DSC, comparing the melting endothermic peak of the drug and the shift of this thermal event to lower temperature in the loaded nanofibers. The absence of the melting event in the composite nanofibers indicated that all of the incorporated drug was converted into an amorphous state, thus suggesting the high compatibility of the drug with the polymer. Wang et al. [91] carried out a compatibility study on shellac nanofibers for the colon-sustained release drug delivery. The fibers contained a ratio of 7.5:1.5 shellac gum to ferulic acid. With the aim of XRD and MDSC, the compatibility between the drug and the carrier was determined. The results showed an endothermic peak at 174 °C corresponding to the melting of the pure ferulic acid, while the loaded nanofibers did not showed any melting peak, indicating that the active ingredient (ferulic acid) was in the amorphous state in the fiber. Kommavarapu et al. [92] reported similar results. They studied the solid dispersion of etravirine by the spray-drying technique to enhance the aqueous solubility and dissolution rate, and they observed the melting of etravirine at 265 °C and the absence of melting events in the spray-dried mixtures with copovidone povidone–copovidone. Evidently, the presence of the amorphous state is an indication of the conservation of the physicochemical properties of the compound. Kelleher et al. [93] carried out a comparative study between the hot-melt extrusion and spray drying of compatible monolithic fixed-dose products comprising of hydrochlorothiazide and ramipril for the treatment of hypertension. By means of MDSC, they found that in the spray-dried products, the drugs were in the amorphous state, and this structure was helpful for maintaining the physical stability after 60 days of storage. Ruthesh et al. [94] evaluated the physicochemical properties of high loads of acetaminophen and aspirin in orally disintegrating strips and compared these properties with the loads in commercial tablets. MDSC was employed for evaluating the compatibility of the drugs with the hydroxypropylcellulose strips, comparing the area under the curve of the drug melting, and considering the absence of a degradation peak at higher temperature.

Although it is clear that MDSC may be very helpful in the characterization of several food and pharmaceutical products, it still may present some disadvantages; in other words, sometimes it is not easy to extract meaning from the MDSC results. Boller, Schick, and Wunderlich [95] tested the effect of time, modulated frequency, and storage temperature on the glass transition temperature of polystyrene by MDSC. They found that the Tg temperature was shifted to lower values as the the modulation frequency decreased. However, the lowest modulated temperature amplitude (heating and cooling rates) led to the sharpest glass transitions but had no effect on the Tg temperature. The reversible heat flow thermograms of polystyrene stored at different temperatures showed that the storage history of samples had no effect on the Tg temperature. They concluded that despite the thermal history of the sample, MDSC was accurate in the measurement of Tg temperature on heating. Coleman and Craig [96] reviewed the principles and uses of DSC, emphasizing the advantages of MDSC. They identified some disadvantages of the MDSC technique; for example, in order to satisfy the requirement for at least six modulations throughout the duration of each thermal event, low underlying heating rates are required. These may not always be desirable. This restriction is significant when analyzing sharp transitions. Bottom [97] concluded that with the reversible heat flow curve obtained from the MDSC studies, the Tg of sulfapyridine was observed with much clarity. However, the main disadvantage of this technique is the data analysis and interpretation, which is more difficult than in conventional DSC.

## 5. Conclusions

Differential scanning calorimetry (DSC) and modulated differential scanning calorimetry (MDSC) have emerged as important techniques for industry because of the thermodynamic parameters obtained such as first-order transitions (melting and crystallization temperature) and second-order transitions (glass transition temperature). These parameters are widely used for developing new products, improving existing ones, and testing the effectiveness of technological procedures in the industry. Specifically, MDSC shows some advantages over other techniques employed in the characterization of phase transitions because of the higher resolution, sensitivity, and the capability of distinguishing reversible and non-reversible processes from the total heat flow. In the food industry, DSC and MDSC have been widely used in the study of the denaturation of proteins, carbohydrate stability, and determining first and second-order transitions in lipids. In pharmaceutical products, these techniques have been used in the characterization of solid components and their stability, and they have also been used to test their compatibility.
